# Supplementary figures and images for: The effects of acute hydrogen peroxide exposure on respiratory cilia motility and viability
Source: PeerJ. 2023 Feb 27;11:e14899. doi: 10.7717/peerj.14899 (PMC9979836; doi:10.7717/peerj.14899)

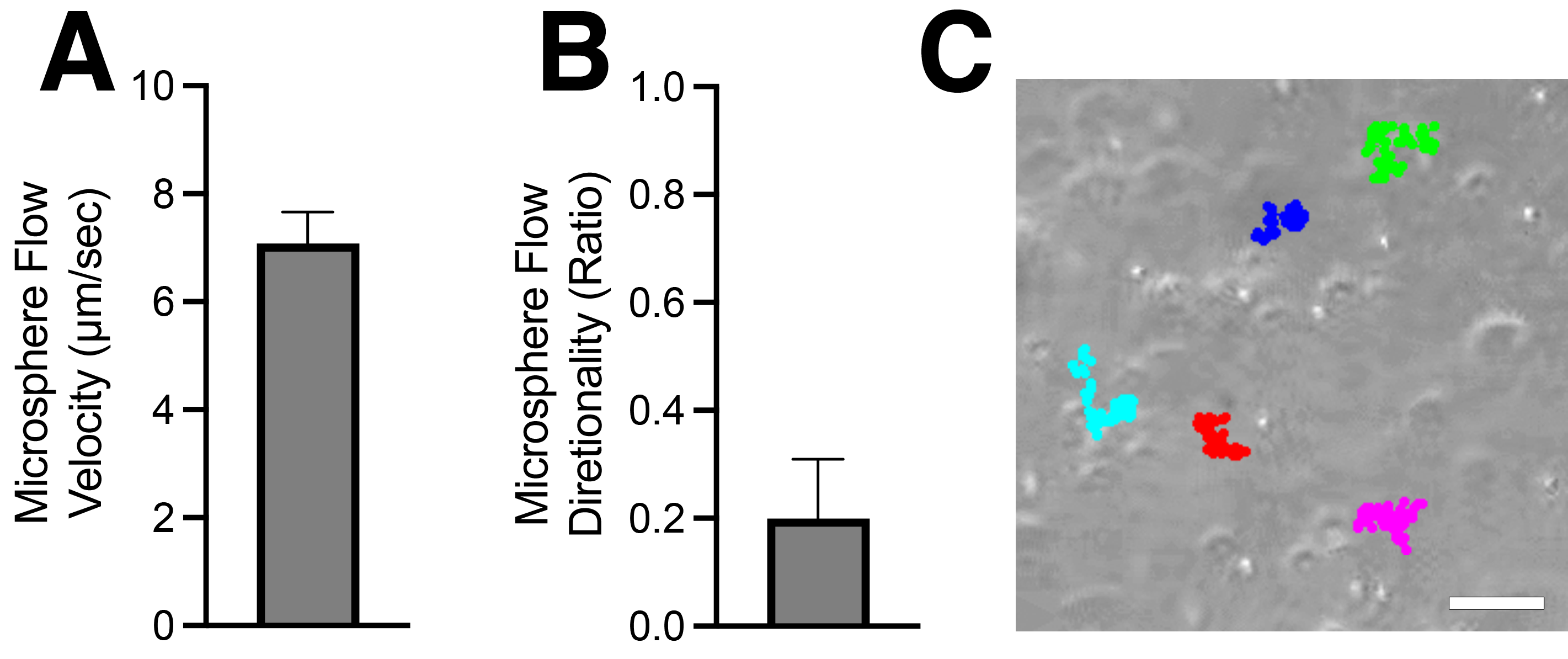

Supplement: Supplemental Information 1 — Microsphere velocity (A) and directionality (B) was calculated for 0.5 µm polystyrene microspheres within L-15 media lacking tracheal tissue to determine Brownian motion values. NB: Directionality was calculated by dividing net microsphere displacement by total distance travelled, microspheres moving in a straight line display directionality ≈1; microspheres moving randomly display directionality ≈0. (C) Representative microsphere tracks highlighting Brownian motion in the absence of cilia generated flow. Data presented as Mean ± SEM (n = 3). Scale bar = 5 µm https://doi.org/10.6084/m9.figshare.19785259 [file peerj-11-14899-s001.png]
