# Supplementary material for: The effects of acute hydrogen peroxide exposure on respiratory cilia motility and viability
Source: PeerJ. 2023 Feb 27;11:e14899. doi: 10.7717/peerj.14899 (PMC9979836; doi:10.7717/peerj.14899)
Supplement: Supplemental Information 2 [file peerj-11-14899-s002.docx]

| Nebulized H_2_O_2_ Claim(s) | H_2_O_2_ Dose/Route | | Treatment time | | Reference URL | |  |
| --- | --- | --- | --- | --- | --- | --- | --- |
| "Nebulized hydrogen peroxide therapy is an inexpensive and simple way to treat most viral respiratory illnesses"  "can be anticipated to eliminate eventual fatal disease outcomes in all but the most advanced cases" | | Household 3% H_2_O_2_ (or diluted), nebulizer with a face mask that covers mouth and nose | | 10-15 minutes, four times a day, until symptoms are relieved | | \| <https://archive.ph/Eq1iy> \| \| --- \| | |
| "As it is a completely non-toxic therapy, nebulisation can be administered as often as desired."  H_2_O_2_ inactivates "dangerous infectious viruses"  "Don’t wait for initial symptoms. Just nebulise at your first opportunity" | | Common household H_2_O_2_ diluted to 3%, nebulizer that covers mouth and nose and emits a fine mist | | begin treatment at first signs of symptoms 10-15 mins four times a day | | \| <https://archive.ph/IUopG> \| \| --- \| | |
| H_2_O_2_ nebulizer treatment ten minutes every waking hour cured flu within 72 hours  Used to treat "hundreds of cases of colds, flus, sinusitis, and bronchitis all with the same great results." | | Nebulized H_2_O_2_, dose not listed | | ten minutes every waking hour | | \| <https://archive.ph/fXjdK> \| \| --- \| | |
| "H_2_O_2_ has many functions, but one of its greatest benefits is aiding the body in ridding viruses."[sic]  "These treatments can be used preventively as well. Daily H_2_O_2_ nebulization therapy may be helpful at stopping even low levels of viral growth, perhaps even undetected by the body." | | Nebulized 3% H_2_O_2_ (full strength or diluted) | | If symptoms present, then 10 min sessions "4 times a day minimally" until feeling better. | | \| <https://archive.ph/zPanN> \| \| --- \| | |
| "Nebulization of hydrogen peroxide the solution for colds, flus and respiratory infections"  "Our favorite substance to nebulizes [sic] is hydrogen peroxide…. because it is extremely safe" | | Nebulized H_2_O_2_, dose not listed | | Treatment time not listed | | \| <https://archive.ph/aNh69> \| \| --- \| | |
| "At home treatment that can cure any virus, including coronavirus"  "As it is a completely non-toxic therapy, nebulization can be administered as often as desired." | | Nebulized 3% H_2_O_2_ | | 10-minute sessions 4 times a day until feeling better | | \| <https://archive.ph/Rc39v> \| \| --- \| | |
| "can help alleviate the symptoms of …. Asthma, Cold and Flu, COVID, Upper Respiratory Infections, Allergy symptoms, Toxic Mold, Chronic Infections" | | Nebulized 3% H_2_O_2_ with magnesium sulfate | | 10 to 15 minutes | | \| <https://archive.ph/SEQpa> \| \| --- \| | |

Supplementary Table 1. Selected online complementary and alternative medicine (CAM) references that recommend nebulized H_2_O_2_ to treat disease, including suggested H_2_O_2_ dose, route, and treatment time.
